# A Path Towards Reproductive Justice: Incorporating a RJ Framework into North Carolina’s Improving Community Outcomes for Maternal and Child Health Initiative

**DOI:** 10.1007/s10995-022-03563-7

**Published:** 2022-11-09

**Authors:** Lindsey Yates, Isabel Morgan, Christine Tucker, Cathy Henderson, Tara Owens Shuler, Dorothy Cilenti

**Affiliations:** 1 Department of Maternal and Child Health, Gillings School of Global Public Health, University of North Carolina at Chapel Hill, McGavran-Greenberg Hall, CB#7445, 27599-7445 Chapel Hill, NC USA; 2 Mecklenburg County Public Health, 249 Billingsley Road, 28211 Charlotte, NC USA; 3 Women’s Health Branch, North Carolina Division of Public Health¸2001Health¸2001 Mail Service Center, 27699-2001 Raleigh, NC USA

**Keywords:** Reproductive Justice, Long-Acting Reversible Contraception, Birth Outcomes, North Carolina

## Abstract

**Purpose:**

Long-acting reversible contraception (LARC) is encouraged as a strategy to address racial disparities in birth outcomes. Black woman-led organizations and stakeholders recommend a thoughtful integration of Reproductive Justice for any LARC programs. This paper will describe how one state-funded maternal and child health program reconceptualized an evidence-based strategy (EBS) focused on increasing access to LARC, to a broader strategy that incorporated principles of Reproductive Justice to improve birth outcomes.

**Description:**

In 2016, North Carolina established the Improving Community Outcomes for Maternal and Child Health (ICO4MCH) program. As part of this program, five county health departments were awarded funding to “increase access to LARC”. Noting community partners’ concerns with this strategy, ICO4MCH leadership revised the strategy to focus on using the Reproductive Justice framework to improve utilization of reproductive life planning and access to LARC. Leaders modified the strategy by changing performance measures and scope of work/deliverables required by grantees.

**Assessment:**

Using quarterly reports and focus group data from ICO4MCH grantees, we identified key steps communities have taken to prioritize Reproductive Justice. Key findings include that sites hosted Reproductive Justice trainings for team members and changed language describing family planning services. These activities were tailored to fit community context and existing perceptions about reproductive health services.

**Conclusion:**

The ICO4MCH program was able to modify a LARC EBS to better emphasize Reproductive Justice. Local agencies desiring to shift their LARC programs should include and value feedback from those with lived experience and partner with organizations committed to Reproductive Justice.

## Significance

*What is known on the subject?* Citing evidence about the effectiveness of LARC, state and local health departments continue to work to increase access to LARC as a method to address unintended pregnancies and racial disparities in unintended pregnancies. We encourage these programs to prioritize Reproductive Justice as they provide contraception health services. *What this study adds?* The Improving Community Outcomes for Maternal and Child Health program modified a LARC EBS to incorporate Reproductive Justice. Local agencies seeking to ground their reproductive health initiatives in Reproductive Justice can use this information to improve their programs.

## Purpose

Long-acting reversible contraception, both intrauterine devices and implants, is a highly effective form of contraception. Use of LARC has been shown to be associated with a greater reduction in unintended pregnancies compared to use of other short-acting contraceptive methods (Peipert et al., 2012). As a result of its effectiveness, some stakeholders viewed LARC as a clinical evidenced-based strategy (EBS) that could increase birth spacing and decrease the rate of unintended pregnancies, with an overall benefit of improving health outcomes for women and infants (Blumenthal et al., 2011; Goldthwaite et al., 2015). In addition to reducing the number of unintended pregnancies, findings from the CHOICE project and the Colorado Family Planning Initiative highlighted that LARC use increases when individuals have access to these methods at low or no cost (Peipert et al., 2012; Ricketts et al., 2014).

In light of this evidence, the reproductive health community called for an increase in access to LARC methods (American College of Obstetrics and Gynecology, 2015; Centers for Disease Control and Prevention, 2015). Policy and practice changes soon followed. Under the Affordable Care Act, qualifying health plans were required to cover the cost of LARC devices and procedures (Guttmacher Institute, 2020a). The Association of State and Territorial Health Officials created the Increasing Access to Contraception Learning Community. The purpose of the learning community was to support state and territorial agencies in developing and sharing best practices aimed at improving access to contraception, including LARC. (Association of State and Territorial Health Officials (ASTHO) Maternal and child health, 2020; Kroelinger et al., 2018).

While the call to improve access to LARC grew, a cohort of family planning providers and advocates, led by Black women, in collaboration with Latinx and other women of color grounded in Reproductive Justice and aware of the potential for a resurgence of reproductive coercion, tempered excitement about prioritizing LARC. Researchers, practitioners, and activists advocated for contraceptive counseling for all contraceptive methods, inclusive of LARC, grounded in Reproductive Justice (Brandi & Fuentes, 2020), but limited published information offered a blueprint for best practices to meaningfully incorporate this framework into existing systems of care, specifically in local health departments.

The purpose of this article is to describe how one state-funded maternal and child health program reconceptualized an EBS focused on increasing access to LARC, to a broader strategy that incorporated principles of Reproductive Justice to improve birth outcomes. We describe this program change, and how it was implemented by grantees. This article can inform how health departments and other local agencies can ground their maternal and child health programs in a Reproductive Justice framework.

## Description

### Reproductive Justice and the Tensions of Increasing Access to LARC

Reproductive Justice is the right to bodily autonomy, the right to decide when to have and not have children and the right to parent your children how you desire (Ross & Solinger, 2017; SisterSong Women of Color Reproductive Justice Collective, n.d.). The term Reproductive Justice was originally coined in 1994 by a collective of Black women committed to advancing reproductive rights and social justice (Ross & Solinger, 2017; SisterSong Women of Color Reproductive Justice Collective, n.d.). Intersectionality, a word credited to Kimberlé Crenshaw but developed by generations of women scholars of color, is embedded in the Reproductive Justice framework (Crenshaw, 1989; Ross, 2017). The framework highlights how the intersection of various systems of oppression impact individuals’ reproductive health decision making. Since its creation, advocates have encouraged the use of the Reproductive Justice framework to ensure equitable and patient-centered access to reproductive health services.

In applying the Reproductive Justice framework to an EBS aimed at increasing access to LARC, stakeholders noted some key issues. First, Reproductive Justice challenges the perception of LARC as a solution to unintended pregnancies. The reproductive health community embraced LARC as a strategy to counter pregnancies that may lead to worse health outcomes for children and women. It has also been proposed as a method to reduce racial disparities in unintended pregnancies (Parks & Peipert, 2016) Higgins (2014) notes that in making this assumption, it “suggests that *lack of access to effective contraceptives* is the primary driver behind this health disparity – and that unintended pregnancies are a *cause* rather than a *consequence* of social inequality”. Reproductive Justice asserts that disparities in reproductive health outcomes are rooted in systematic racism and other forms of oppression. By not acknowledging the racist and inequitable settings that contributes to these outcomes, a LARC-focused strategy is insufficient.

Second, Reproductive Justice seeks to ensure that people have access to the reproductive services they want. Although some people may desire LARC, a primary focus on this method, without ensuring access to other methods or services, feels reminiscent of reproductive injustices experienced by disenfranchised communities. One well known example is the eugenics movement in the US during the 20th century, resulting in the coerced and forced sterilizations of individuals, including 7,000 people in North Carolina (Schoen, 2001). The impact of this history remains in the collective memory of historically racialized and marginalized communities that disproportionately experienced sterilizations, including Black, Indigenous, and Latinx people, and individuals with developmental disabilities.

Lastly, Reproductive Justice requires that individuals have autonomy, including the choice to have LARC devices removed when they want (Higgins, 2014). When a LARC device no longer meets an individual’s needs, it should be removed without consequence or shame. The primary focus on LARC insertion, instead of LARC services, is in part the result of research aimed at increasing LARC users. An EBS that is informed by Reproductive Justice must also include increasing access to LARC removal.

### Improving Community Outcomes for Maternal and Child Health and LARC

The North Carolina General Assembly legislated funding (Session Law 2015 − 241) to invest in evidence-based programs shown to reduce infant mortality and improve the birth and health outcomes for children from birth to five years of age (Morgan et al., 2020). In fiscal year 2016, North Carolina launched the Improving Community Outcomes for Maternal and Child Health (ICO4MCH) program to provide local health departments with resources and infrastructure to address these aims. Five local health departments (herein referred to as sites) were awarded funding, covering 14 counties across the state. These sites include both urban and rural counties, serving different populations, but having high rates of adverse birth outcomes.

In the ICO4MCH program, explained in detail elsewhere (Morgan et al., 2020), all sites were required to implement an EBS focused on increasing access to LARC. The LARC EBS focused on ensuring that individuals of childbearing age could access LARC in their communities. The EBS was based on best practice recommendations developed from the ASTHO Learning Community (Association of State and Territorial Health Officials (ASTHO) Maternal and child health, 2020) and the Colorado Family Planning Initiative (Colorado Department of Public Health and Environment, 2017; Kelly et al., 2019). Sites were required to complete a variety of activities aimed at increasing access to LARC including provider training about LARC placement, community education and outreach about LARC, and helping agencies develop and sustain same-day LARC placement services. Sites created implementation teams (IT) and community action teams (CAT) to guide the implementation of all EBS, including the LARC EBS. These teams included people with lived experience, health and human services staff members, public health professionals and other stakeholders invested in improving birth outcomes.

In fiscal year 2017, one urban ICO4MCH site expressed concerns about implementing a strategy aimed at increasing access to LARC to improve birth outcomes. Individuals with lived experience and other local partners noted that focusing on LARC would create actual or perceived reproductive coercion. They recognized that poor practices of the health care system, including a recent systematic failure to ensure timely and quality reproductive health care (Clasen-Kelly, 2017), created mistrust amongst the community. They recommended a strategy more focused on Reproductive Life Planning and informed consent. The site shared local concerns with state stakeholders who researched options and recommended the Reproductive Justice framework.

### Reconceptualizing a LARC Evidence Based Strategy

To address concerns of reproductive coercion, ICO4MCH leaders made iterative changes to modify the EBS so it was better focused on Reproductive Justice, while also working to ensure access to LARC for individuals who desired this method. To modify the EBS ICO4MCH leaders consulted with various stakeholders. They engaged with SisterSong Women of Color Reproductive Justice Collective (herein referred to as SisterSong). SisterSong provided their Reproductive Justice 101 training to all ICO4MCH staff (approximately 50 people across all sites). During these trainings participants learned about the history of Reproductive Justice, definitions of reproductive oppression, and how intersectionality influences reproductive decision making. In addition to training, leaders met monthly with site coordinators to discuss challenges with the EBS and ideas for modification. They also worked with evaluators to identify evaluation measures that would allow them to monitor change, and with implementation specialists to problem solve and strategize about implementation challenges of the EBS.

As a result of these discussions ICO4MCH leaders modified the EBS by changing the performance measures and scope of work/deliverables required of sites. A list of the changes to performance measures are outlined in Table 1. Two notable changes were made. First, leaders removed a 10% target increase in the number of LARC users. Instead, sites were encouraged to center increasing access to all contraceptive methods available to individuals, focusing on family planning access, not a specific method. Second, leaders focused on training patients, community members, and providers about Reproductive Justice including education about patient-centered contraception counseling. The modified EBS was renamed “Using a Reproductive Justice framework to improve utilization of reproductive life planning and access to Long-Acting Reversible Contraception”. The name of the EBS continued to include “LARC” but added “Reproductive Justice” and “Reproductive Life Planning”. Leaders choose this name to reflect multiple, simultaneous goals of the updated strategy. Program leaders wanted to prioritize principles of Reproductive Justice in all discussions about parenting and birthing plans and ensure that individuals who desired LARC or LARC services could access them in their communities if these methods aligned with their reproductive plans.

Although ICO4MCH leaders made changes to the EBS, the extent to which the modification influenced sites’ ability to implement the EBS was unclear.

**Table 1:** Key Performance Measures Before and After Reconceptualization of a LARC Evidence Based Strategy

Before	How was this performance measure changed?
Increase the number of clients who receive a LARC​	This performance measure was removed and replaced with: Increase the number of local health department (LHD) patients who report access to all methods without pressure from providers.
Increase men/women reached via LARC education and outreach events	This performance measure was modified to specify that education and outreach efforts use a reproductive justice framework when discussing family planning methods, specifically LARCs, reproductive life planning (RLP), a tiered approach to contraceptive counseling including, potential side effects, and informed consent of family planning methods, specifically, LARCs.
Decrease health care providers perceived barriers to LARC utilization	This performance measure was removed
Increase the # of local health departments and community providers who offer LARC3​	This performance measure was replaced with: Increase the percent of LHD providers who utilize the RLP protocol when providing health care services to women in all LHD clinics (family planning, maternal health, etc.)
Increase the number of local health departments (HD) who have a same-day insertion policy​	No change to this measure
Increase the number of local HD and community providers who offer same-day insertion​	No change to this measure
Increase the number of local HD and community providers participating in family planning training	No change to this measure

## Assessment

### Methods and Materials

We used focus group data and quarterly report data to assess how sites implemented the modified EBS. Program evaluators conducted focus groups with each of the five sites at the end of fiscal year 2019 (approximately one year after modifying the EBS). The purpose of the focus groups was to assess process, successes, and challenges of the teams, including the teams’ work on the modified strategy. Thirty -seven individuals participated in the focus groups, with 5–10 participants at each site. Half (20) of the participants identified as members of both the CAT and the IT. Most participants (27) were LHD staff. More than half (21) of the participants had been involved in the CAT/IT for one year or longer. Evaluators used thematic analysis to analyze transcripts from the focus groups and summarize key findings.

Quarterly report data from fiscal year 2019 through fiscal year 2020 detailed activities and accomplishments related to various performance measures for each site. The information is entered electronically into a REDCap database by site coordinators and other site staff. This information is used to provide feedback to sites as they progress, and to create the annual evaluation report. For the reconceptualized EBS, sites responded to 18 questions about their activities. These included three open-ended questions such as one that read, “During the last quarter, how did you incorporate the Reproductive Justice framework into your work?”

This project was a program evaluation and deemed not to be human subjects research by the Institutional Review Board at the University of North Carolina at Chapel Hill (IRB #16-2538), therefore not requiring written or signed consent on behalf of participants.

Each of the five sites adopted new actions to incorporate Reproductive Justice into their work. Below we outline findings from the focus groups and quarterly reports that describe their efforts to adjust to the modified strategy. Illustrative quotes that provide greater detail about various implementation strategies are available in Tables 2, 3 and 4.

**Table 2:** Illustrative Quotes from Focus Group and Quarterly Report data about Providing Reproductive Justice Training

*[The site coordinator] gave a training on Reproductive Justice to providers at [a local teaching hospital].* *Urban Site*
*We have begun to approach the reproductive justice framework from a uniquely [regional] perspective by thinking of [regional] cultural competency as a whole. We have spent this quarter continuing our engagements with consulting agency [Information redacted for confidentiality] who is crafting a workshop for our Regional Leadership Team around [regional] cultural competency. This workshop will include elements of the Reproductive Justice framework, as it pertains to [this region]. Because our regional demographics are majority white with very little diversity, our discussions around Reproductive Justice will be uniquely focused on elements of reproductive justice that apply in [this region], such as reproductive autonomy and coercion issues, which are widespread and prevalent. In addition to preparing for this workshop, we have also been exploring our options for furthering the Reproductive Justice framework in our counties. We have connected with two reproductive justice consulting organizations: SisterSong and SisterReach. We already knew of SisterSong’s work through the Reproductive Justice 101 training our team attended in the spring, but came across SisterReach while looking for other options. SisterReach is based out of rural Tennessee, and does similar work to that of SisterSong. However, SisterReach may provide us an RJ experience tailored to our rural population, since their organization has expertise in implementing the RJ framework in rural communities, rather than urban and largely minority. We plan to continue consulting with these two agencies in conjunction with our CAT teams to determine the best fit for our site.* Rural Site
*We educated staff on the reproductive justice framework. We are reviewing clinic practices around conversations with clients to ensure they honor Reproductive Justice Framework principles. We’ve also openly discussed the challenges of Tiered Counseling and provider/worker coercion.* Urban Site
*Reviewed Reproductive Justice Framework with internal and external providers along with our clinical staff. Our collaborative also coordinated with SisterSong to provide Reproductive Justice 101 and 102 to Local Health Department staff in both counties and providers in [another] County.* Rural Site
*During Quarter 3, the [our] Collaborative collaborated with the [another collaborative in] ICO4MCH to bring the Sister Song Reproductive Justice Framework 101 training to all 6 counties and 4 additional counties/organizations. There were 28 individuals that successfully completed the training, learning how to utilize the Reproductive Justice Framework within their organization and the work that they do.* Rural/Urban Site

**Table 3:** Illustrative Quotes from Focus Group and Quarterly Report data about Reframing Language

*We also make sure to always tell people in the community that we’re working to increase people’s access to whatever type of birth control works for them, not a particular method over others.* Urban Site
*…We spent a lot of time thinking about wording and ensuring our clinic is offering inclusive support and counseling, and comprehensive contraception options to patients free of judgement or coercion.* Rural Site
*The Reproductive Justice framework was tied into all presentations and discussions with partners. The reinforcement of providing equal and accessible services to all clients is included in all power points and highlighted in educational outreach.* Urban Site
*During Quarter 4 the Reproductive Justice Framework was utilized in each of our clinics when counseling patients. Our clinic coordinators and providers have spoken on the methods in which they counsel their patients, reminding them that it is their body, their choice, and educating them on the different contraceptive methods each clinic has to offer, as well as the importance of a reproductive life plan and topics such as birth spacing and folic acid.* Rural/Urban Site

**Table 4:** Illustrative Quotes from Focus Group and Quarterly Report data about Challenges Implementing Reproductive Justice

*Then the history of forced sterilization in North Carolina it clearly yes that could be some very deep mistrust and suspicion of any government-supported programs promising long-term birth control.* Urban Site
*But then that being said another boulder [challenge] is that with a rural community it’s hard to find groups. Like not only are there not a lot of providers but they’re also, not a lot of just existing groups of people that will like come and listen to this kind of thing. So it’s hard to get a captive audience.* Rural Site
*Keeping the team members engaged once you get them and that goes along with identifying – first identifying stakeholders and people that are going to be at the table and then once they get to the table, how do we keep them there.”* Urban Site
*I think, the negative stigma or the stigma because – sometimes it’s positive, I guess I should say preconceived messages – messaging around it, not necessarily stigma because when you say reproductive life planning, it’s like oh yeah, birth control, you can get them. What about people who don’t want birth control and that – so part of it is the reproductive life plan.* Rural Site
*There’s a stigma around [the modified] strategy that people think it’s just sex, and it’s become taboo. It’s like oh, I don’t need that. I don’t want kids, and it’s like ok here’s an IUD/Implant brochure…* Rural/Urban Site

## Findings

### Providing Reproductive Justice Training

Sites hosted or supported Reproductive Justice trainings for local staff and clinicians. Sites did not provide information about the content and format of the trainings, however, they described that trainings were led by facilitators skilled in Reproductive Justice. Some sites chose to partner with organizations like SisterSong (https://www.sistersong.net/) or SisterReach (https://www.sisterreach-tn.org/), while others had their own staff familiar with Reproductive Justice present on the principles of the framework. Site leaders noted that community composition and context was the primary reason why some partnered with certain organizations, while others did not. Many sites prioritized training providers and health department staff in FY2019, with refresher trainings available throughout the fiscal year for current and new staff or providers. From fiscal year 2019 through fiscal year 2020, 444 providers and staff across all sites were trained in Reproductive Justice and counseling approaches relevant to reproductive life planning.

### Reframing Language

Sites worked to ensure that the language used to describe family planning services incorporated Reproductive Justice principles. The structure and format of the information varied based on the audience and community context. In both written and oral communication with patients and providers, sites used plain language that centered patient autonomy when considering their family planning and reproductive health needs. Information presented to more general audiences focused on patient autonomy and reproductive life planning, including the identification of key terms and phrases perceived as appropriate by their communities and partners. Considering such language enabled leaders to effectively communicate the principles of Reproductive Justice to various groups, helping to gain stakeholder buy-in.

### Challenges with Implementing Reproductive Justice

Sites leaders noted that they managed various challenges as they worked to incorporate Reproductive Justice into existing care models. Some of the challenges included community distrust of a state-funded program, keeping multiple stakeholders engaged long-term, and challenges identifying groups to train because of a lack of available providers or because of community reluctance to discuss topics related to contraception health services. Considering these issues, sites made changes based on the context of their communities.

## Conclusion

In recent years advocates have encouraged leaders responsible for LARC and family planning programs to incorporate Reproductive Justice into these programs (Cappello, 2021; SisterSong Women of Color Reproductive Justice Collective and National Women’s Health Network, n.d.), but there is less information about how state and local agencies have attempted to make such changes. The work done by ICO4MCH leaders and local staff offers valuable insight about incorporating Reproductive Justice into state-funded programs. Leaders in other communities who are invested in making a similar change must seek out and apply feedback from local grantees and people with lived experience, partner with organizations at the helm of the Reproductive Justice movement, and center Reproductive Justice in communication and measurement.

Although there is little information about changes at the state level, the New York City Department of Health and Mental Hygiene (DOHMH) has documented some of their work to center Reproductive Justice. In 2015 DOHMH launched the “Maybe IUD” campaign. Similar to North Carolina, New York leaders received feedback from key stakeholders about possible coercive practices that may arise as a result of the campaign (Roberts et al., 2016). Like ICO4MCH leaders, DOHMH leaders partnered with Reproductive Justice organizations, and committed to rebranding the campaign to focus on access to all contraceptive methods (Gan, 2015). There is less information about the longer-term implications of this change. Future research can compare population health outcomes among states and municipalities working to prioritize Reproductive Justice compared to those who are not.

### Limitations

We recognize that there are important limitations to these efforts. Since the modification of the EBS, patient or community member perceptions of the change to incorporate Reproductive Justice in the reproductive health services available in funded sites have not been captured. Community voice and client satisfaction with services are a vital component of verifying that local health department and partnering clinics are practicing Reproductive Justice principles. Sites were encouraged to collect feedback from clients in the coming year. One rural site is also using the Person-Centered Counseling Measure (https://pcccmeasure.ucsf.edu/) to better assess consumer experience with contraception services. Further, while over 400 providers were trained in the Reproductive Justice framework, updated information from providers about their perceptions of the utility of Reproductive Justice trainings for their interactions with patients is unavailable. A survey collecting updated information was underway, but then the COVID-19 pandemic began. As a result, survey collection from providers stalled and was eventually suspended. Of note, following the guidelines set by the Hyde Amendment, North Carolina does not use public funds to cover abortion except in cases of maternal life endangerment, rape or incest. As such, ICO4MCH does not fund access to abortion services, which is an important reproductive health service that aligns with the principles of Reproductive Justice (American Civil Liberties Union, n.d.; Guttmacher Institute, 2020b).

### Next Steps

After initially modifying the EBS, ICO4MCH leaders continue to seek input from sites, evaluators, and implementation specialists about the structure and implementation of the modified EBS. Through these ongoing conversations ICO4MCH leaders learned about the change’s sites were able to make, and some of the challenges sites faced as they worked on this strategy. Based on this feedback ICO4MCH leaders made additional changes to the EBS, including renaming the strategy “Using a Reproductive Justice framework to improve utilization of reproductive life planning” in fiscal year 2021. Program leaders will continue to seek input about the EBS from relevant stakeholders to strengthen the EBS and ensure equitable, patient-centered access to family planning services.

As health care, public health, and social service agencies recognize the relevance of Reproductive Justice, finding ways to meaningfully incorporate this framework into their existing systems of care is growing more relevant. For state and local health departments, these findings may inform the implementation of maternal and child health programs guided by Reproductive Justice principles.

## Data Availability

Data is available upon request and with the approval of the Women’s Health Branch, North Carolina Department of Health and Human Services.

## References

[CR1] American Civil Liberties Union. (n.d.). *Public funding for abortion.* Retrieved January 20 (2021). from https://www.aclu.org/other/public-funding-abortion

[CR2] American College of Obstetrics and Gynecology (2015). *Increasing access to contraceptive implants and intrauterine devices to reduce unintended pregnancy. Committee Opinion No. 642.* http://www.acog.org/Resources-And-Publications/Committee-Opinions/Committee-on-Gynecologic-Practice/Increasing-Access-to-Contraceptive-Implants-and-Intrauterine-Devices-to-Reduce-Unintended-Pregnancy#5 10.1097/AOG.0000000000001106 26393458

[CR3] Association of State and Territorial Health Officials (ASTHO) Maternal and child health. (2020). *Increasing Acces to Contraception: LARC Learning Community Presentations*. https://www.astho.org/Programs/Maternal-and-Child-Health/Long-Acting-Reversible-Contraception-LARC/Learning-Community-Presentations/

[CR4] Blumenthal PD, Voedisch A, Gemzell-Danielsson K (2011). Strategies to prevent unintended pregnancy: Increasing use of long-acting reversible contraception. Human Reproduction Update.

[CR5] Brandi K, Fuentes L (2020). The history of tiered-effectiveness contraceptive counseling and the importance of patient-centered family planning care. American Journal of Obstetrics and Gynecology.

[CR6] Cappello, O. (2021). *Powerful Contraception, Complicated Programs: Preventing Coercive Promotion of Long-Acting Reversible Contraceptives* (Vol. 24). Guttmacher Policy Review. https://www.guttmacher.org/sites/default/files/article_files/gpr2403621.pdf

[CR7] Centers for Disease Control and Prevention (2015). *The 6|18 Initiative Evidence Summary Prevent Unintended Pregnancy*. https://www.cdc.gov/sixeighteen/docs/6-18-evidence-summary-pregnancy.pdf

[CR8] Colorado Department of Public Health and Environment (2017). *Taking the Unintended Out of Pregnancy: Colorado’s Success with Long-Acting Reversible Contraception*,. www.colorado.gov/cdphe/cfpi-report

[CR9] Crenshaw, K. (1989). *Demarginalizing the intersection of race and sex: A Black feminist critique of antidiscrimination doctrine, feminist theory, and antiracist politics [1989]*. *1*(8). https://chicagounbound.uchicago.edu/uclf/vol1989/iss1/8

[CR10] Clasen-Kelly, F. (2017, June 17). C. M. and E. M. Nurse sounded alarm over risk to patients: Problem ‘much worse’ than public was told. *The Charlotte Observer*. https://www.charlotteobserver.com/news/local/article158797189.html

[CR11] Gan, V. (2015, October 7). *Why the City of New York wants to talk to you about IUDs*. Bloomberg CityLab. https://www.bloomberg.com/news/articles/2015-10-07/new-york-city-health-department-launches-maybe-the-iud-contraceptive-awareness-initiative

[CR12] Goldthwaite LM, Duca L, Johnson RK, Ostendorf D, Sheeder J (2015). Adverse Birth Outcomes in Colorado: Assessing the Impact of a Statewide Initiative to Prevent Unintended Pregnancy. American Journal of Public Health.

[CR13] Guttmacher Institute (2020a). *Insurance Coverage of Contraceptives*. https://www.guttmacher.org/state-policy/explore/insurance-coverage-contraceptives

[CR14] Guttmacher Institute (2020b). *State Facts About Abortion: North Carolina*. https://www.guttmacher.org/fact-sheet/state-facts-about-abortion-north-carolina

[CR15] Higgins JA (2014). Celebration Meets Caution: Long Acting Reversible Contraception (LARC)’s Boons, Potential Busts, and the Benefits of a Reproductive Justice Approach. Contraception.

[CR16] Kelly, A. M., Lindo, J. M., & Packham, A. (2019). *The Power of the IUD: Effects of Expanding Access to Contraception Through Title X Clinics* (NBER Working Paper No. 25656; NBER WORKING PAPER SERIES). https://www.nber.org/system/files/working_papers/w25656/w25656.pdf

[CR17] Kroelinger, C. D., Morgan, I. A., DeSisto, C. L., Estrich, C., Waddell, L. F., Mackie, C., Pliska, E., Goodman, D. A., Cox, S., Velonis, A., & Rankin, K. M. (2018). State-Identified Implementation Strategies to Increase Uptake of Immediate Postpartum Long-Acting Reversible Contraception Policies. *Journal of Women’s Health (2002)*. 10.1089/jwh.2018.7083 10.1089/jwh.2018.7083 PMC6420383 30388052

[CR18] Morgan IA, Tucker C, Urlaub DM, Shuler TO, Cilenti D (2020). Leveraging an Academic-Practice Partnership to Improve Maternal and Child Health Outcomes in North Carolina. North Carolina Medical Journal.

[CR19] Parks C, Peipert JF (2016). Eliminating health disparities in unintended pregnancy with long-acting reversible contraception (LARC). American Journal of Obstetrics and Gynecology.

[CR20] Peipert JF, Madden T, Allsworth JE, Secura GM (2012). Preventing Unintended Pregnancies by Providing No-Cost Contraception. Obstetrics & Gynecology.

[CR21] Ricketts S, Klingler G, Schwalberg R (2014). Game change in Colorado: widespread use of long-acting reversible contraceptives and rapid decline in births among young, low-income women. Perspectives on Sexual and Reproductive Health.

[CR22] Roberts, L., Kaplan, D., & Hygiene, S. (2016). and R. J. C. E. G. and the N. Y. C. D. of H. and M. Locating LARC Within the Context of Sexual and Reproductive Justice. *American Journal of Public Health*, *106*(7), e13–e14. 10.2105/AJPH.2016.303206 10.2105/AJPH.2016.303206 PMC4984769 27285266

[CR23] Ross LJ (2017). Reproductive Justice as Intersectional Feminist Activism. Souls.

[CR24] Ross, L., & Solinger, R. (2017). *Reproductive Justice (An Introduction) (First Edit)*. University of California Press

[CR25] Schoen J (2001). Between Choice and Coercion: Women and the Politics of Sterilization in North Carolina, 1929–1975. Ournal of Women’s History.

[CR26] SisterSong Women of Color Reproductive Justice Collective. (n.d.). *Reproductive Justice*. Retrieved October 8 (2020). from https://www.sistersong.net/reproductive-justice

[CR27] SisterSong Women of Color Reproductive Justice Collective and National Women’s Health Network. (n.d.). *Long-Acting Reversible Contraception Statement of Principles*. Retrieved May 31 (2022). from https://nwhn.org/wp-content/uploads/2019/03/LARC-Statement-of-Principles-1-1.pdf

